# Identification of new benzofuran derivatives as STING agonists with broad-spectrum antiviral activity

**DOI:** 10.1016/j.virusres.2024.199432

**Published:** 2024-07-08

**Authors:** A. Paulis, A. Onali, P.O. Vidalain, V. Lotteau, C. Jaquemin, A. Corona, S. Distinto, G.L. Delogu, E. Tramontano

**Affiliations:** a Department of Life and Environmental Sciences, University of Cagliari, Monserrato 09042, Italy; b CIRI, Centre International de Recherche en Infectiologie, University Lyon, Inserm, U1111, Université Claude Bernard Lyon 1, CNRS, UMR5308, ENS de Lyon, Lyon F-69007, France

**Keywords:** STING, Broad-spectrum antiviral, Immune modulation, Benzofurans, Human coronaviruses

## Abstract

The Stimulator of Interferon Genes (STING) is involved in cytosolic DNA sensing and type I Interferons (IFN-I) induction. Aiming to identify new STING agonists with antiviral activity and given the known biological activity of benzothiazole and benzimidazole derivatives, a series of benzofuran derivatives were tested for their ability to act as STING agonists, induce IFN-I and inhibit viral replication. Compounds were firstly evaluated in a gene reporter assay measuring luciferase activity driven by the human IFN-β promoter in cells expressing exogenous STING (HEK293T). Seven of them were able to induce IFN-β transcription while no induction of the IFN promoter was observed in the presence of a mutated and inactive STING, showing specific protein-ligand interaction. Docking studies were performed to predict their putative binding mode. The best hit compounds were then tested on human coronavirus 229E replication in BEAS-2B and MRC-5 cells and three derivatives showed EC_50_ values in the μM range. Such compounds were also tested on SARS-CoV-2 replication in BEAS-2B cells and in Calu-3 showing they can inhibit SARS-CoV-2 replication at nanomolar concentrations. To further confirm their IFN-dependent antiviral activity, compounds were tested to verify their effect on phospho-IRF3 nuclear localization, that was found to be induced by benzofuran derivatives, and SARS-CoV-2 replication in Vero E6 cells, lacking IFN production, founding them to be inactive. In conclusion, we identified benzofurans as STING-dependent immunostimulatory compounds and host-targeting inhibitors of coronaviruses representing a novel chemical scaffold for the development of broad-spectrum antivirals.

## Highlights

• Benzofuran derivatives were shown to induce IFN-I expression in a STING-dependent luciferase assay.
• Activity as STING agonist was confirmed by mutagenesis studies.
• Antiviral effect of BZFs was demonstrated on HCoV-229E and SARS-CoV-2 replication.
• IFN-I mediated antiviral effect was confirmed by immunofluorescent analysis.

## Glossary

2′3′-cGAMP: 2′3′-cyclic GMP-AMP
BZFs: benzofurans
CDNs: cyclic dinucleotides
cGAS: cyclic GMP-AMP synthase
di-ABZIs: dimeric amidobenzimidazoles
HBV: Hepatitis B Virus
HCoVs: Human Coronaviruses
HSV-2: Herpes Simplex Virus 2
IFN-I: Type I Tnterferons
p/TBK1: phosphorylated / Tank Binding Kinase 1
SARS-CoV-2: Severe Acute Respiratory Syndrome Coronavirus 2
SAR: Structure Activity Relationship
STING: Stimulator of Interferon Genes

## 1 Introduction

The cyclic GMP-AMP synthase (cGAS) - Stimulator of Interferon Genes (STING) pathway is a major line of defense against viral and bacterial infections and tumor onset (Barber, 2015; Burdette et al., 2011; Cai et al., 2020; Li et al., 2017, 2020; Paulis and Tramontano, 2023; Woo et al., 2014a). STING activation occurs in response to either exogenous or endogenous cyclic dinucleotides (CDNs) such as 2′3′-cyclic GMP-AMP (2′3′-cGAMP). Downstream dsDNA detection the cGAS receptor produces 2′3′-cGAMP (Cai et al., 2014; Li et al., 2016; Unterholzner and Dunphy, 2019a; Xia et al., 2016) that selectively binds STING homodimers triggering type I interferons (IFN-I) production (Deng et al., 2014a; Ishikawa et al., 2009). STING monomers are anchored to endoplasmic reticulum's membrane through transmembrane domain, where STING interacts with TANK Binding Kinase 1 (TBK1) (Dobbs et al., 2015; Ishikawa et al., 2009; Shu et al., 2012; Sun et al., 2009). Following STING conformational changes induced upon CDNs binding, TBK1 undergoes trans-phosphorylation and, in turn, phosphorylated TBK1 (pTBK1) phosphorylates Interferon Regulatory Factor 3 (IRF3) that dimerizes and translocates into the nucleus leading to IFN-I transcription, activating the antiviral response (Seth et al., 2005; Zhang et al., 2019; Zhong et al., 2008).

During the last decade, the STING pathway has been explored as possible druggable target for the development of broad-spectrum antivirals (Cavlar et al., 2013; Cerón et al., 2019; Corrales et al., 2015; Liu et al., 2017; Shu et al., 2012; Weiss et al., 2017; Zhu et al., 2020). The natural STING ligand 2′3’ cGAMP has been tested for its ability to inhibit Herpes Simplex Virus 2 (HSV-2) replication, showing strong IFN-I induction and potent antiviral effect, both *in vitro* and in animal model (Cai et al., 2020; Skouboe et al., 2018; Su et al., 2022); however poor permeability and metabolic instability were observed, suggesting its inadequate chemical properties for drug therapy (Berger and Lawler, 2018; Liu et al., 2017). Another explored STING ligand was the antitumor drug DMXAA that was shown to inhibit viral replication in mice and murine models, but it was unable to inhibit viral replication in human derived cells, due to its selectivity for mouse STING (Jameson et al., 2003; Kim et al., 2013). Among natural compounds, the antimicrobial xantone Alpha-Mangostin, was found to induce IFN-I and to inhibit Dengue Fever Virus (DENV) and Hepatitis B Virus (HBV) replication in cell-based assays, while no antiviral activity has been reported in animal models (Cavlar et al., 2013; Choi et al., 2014; Kim et al., 2013; Panda et al., 2021; Tarasuk et al., 2022; Yongpitakwattana et al., 2021) Lately, a benzothiophene derivative, MSA-2, has been reported as STING agonist with antitumor properties (Pan et al., 2020) and the dimeric amidobenzimidazoles (di-ABZIs) were identified through in silico studies as potential STING agonists, capable of inducing IFN-I production, chemokine CXCL1 and interleukin 6 (IL-6) transcription. In particular, two derivatives, di-ABZI-3 and di-ABZI-4, showed antiviral properties against parainfluenza 3, rhinovirus, human coronaviruses (HCoVs) OC43 and severe acute respiratory syndrome coronavirus 2 (SARS-CoV-2) (Liu et al., 2021; Zhu et al., 2021, 2020). More recently, cGAMP and di-ABZI were reported to successfully inhibit Coxsackievirus B3 (CVB3) replication in HeLa cells (Mohamud et al., 2024).

Hence, STING agonists are host-targeting molecules inducing innate immunity with potentially broad-spectrum antiviral activity. To identify novel antiviral agents and given the reported STING-agonist activity of benzothiophene (Pan et al., 2020) and benzimidazole derivatives (Zhu et al., 2021), we studied the activity of a new series of benzofurans derivatives (BZFs), whose scaffold is a bioisostere of both benzothiophene and benzimidazole substructures (Barillari and Brown, 2012; Brown, 2012). Furthermore, BZF is a common moiety present in many biologically active natural and therapeutic compounds representing a suitable scaffold for the development of novel bioactive molecules (Duncan et al., 2021; Khanam and Shamsuzzaman, 2015; Miao et al., 2019; Naik et al., 2015; Nevagi et al., 2015; Pan et al., 2020; Xu et al., 2019). Hence, thirteen in house BZF derivatives bearing different substituents were selected (Delogu et al., 2022, 2021, 2016), and subjected to biological assay to assess their ability to induce IFN and to inhibit viral replication.

## 2 Materials and methods

### 2.1 Synthesis

Methoxylated 2-phenylbenzofurans and hydroxylated 2-phenylbenzofurans were obtained following the procedures reported in previous studies (Delogu et al., 2022, 2021, 2016).

### 2.2 Cells and reagents

HEK293T cells (ATCCⓇ CRL-3216™) were maintained in Dulbecco's modified Eagle's medium (DMEM; Gibco), 10 % v/v fetal bovine serum (FBS; Gibco) and 1x Pen-strep (Euroclone). BEAS-2B cells (Pierre-Olivier Vidalain) were maintained in DMEM/F-12 (Gibco), 5 % v/v FBS HI (Gibco), 1 % Kanamycin (Thermo-Fisher Scientifics). Vero-E6 GFP cells (Janssen Pharmaceutical) were maintained in DMEM (Gibco) supplemented with 10 % v/v FBS heat inactivated (HI) (Gibco), 0.075 % Na bicarbonate (7.5 % solution, Gibco), and 1x Pen-strep (Euroclone). Calu-3 cells (ATCC^Ⓡ^ HTB-55™) were maintained in DMEM, 10 % v/v FBS HI (Gibco), 1x Pen-strep (Euroclone), 1 mM Na Pyruvate (Euroclone), 1 mM Essential Amino Acids (Euroclone). MRC-5 cells (ATCC: CCL-171™) were maintained in MEM (Gibco), 10 % v/v FBS HI (Gibco), 1 mM Na Pyruvate (Euroclone), 1 mM Non-Essential Amino Acids (Euroclone) and 1x Pen-strep (Euroclone). All cells were maintained under 5 % CO_2_ at 37 °C.

HCoV-229E (ATCCⓇ VR-740™) was propagated in MRC5 cells; SARS-CoV-2 BetaCoV/Belgium/GHB-03021/2020 strain was kindly provided by KU Leuven. All SARS-CoV-2-related work was carried out in certified, high-containment biosafety level-3 facility at the University of Cagliari.

Plasmid pGL-IFN-β-luc was kindly provided by Prof Stephan Ludwig from the Institute of Molecular Virology, (University of Münster, Germany). pRL-TK from Promega (Promega Italia S.r.l. Milan, Italy). pUNO1-hSTING-HA3x from Invivogen. pDS_X_HA was kindly provided by Pierre-Olivier Vidalain.

### 2.3 Plasmid mutagenesis

The plasmid pUNO1-hSTING-HA3x was mutated with the QuikChange Lightning Site-Directed Mutagenesis Kit (Agilent Technologies) according to manufacturer's indications. Primers used were forward CCG TGC GGA GAG GGA GTT GCT TTT CCA TTC CAC T reverse: AGT GGA ATG GAA AAG CAA CTC CCT CTC CGC ACG G, mutagenesis was confirmed through sequencing.

### 2.4 Gene reporter assay IFNβ induction

HEK293T were seeded at 2*10^4^ cells/well in a white 96-well plate and incubated overnight to reach 90 % confluency. 24 h later, cells were transfected with 60 ng pGL-IFN-β-luc, 10 ng pRL-TK, 10 ng pUNO1-hSTING-HA3x or pDEST-HA or pUNO1-hSTING-HA3xP371Q, using JetPrime transfection reagent (Polyplus). 24 h post transfection, cells were treated with the indicated concentration of compound. Luciferase activity was detected using homemade solutions (Fanunza et al., 2018) and luminescence was read with Victor Nivo5 PerkinElmer. The relative light units (RLU) were normalized against renilla luciferase luminescence as the fold induction over unstimulated controls.

### 2.5 Western blot

HEK293T cells were seeded in 12-well plates at 10^5^ cells per well; 24 h after seeding, cells were treated with the indicated compound concentrations diluted in culture medium. Doxorubicin was used as control of genotoxic effect at 0.5 μM concentration. After 24 h, the cell culture medium was removed, cells were washed with cold Phosphate Buffer Saline (PBS) and proteins were extracted with 200 μL RIPA buffer (0.05 M Tris–HCl, pH 7.4, 0,15 M NaCl, 0,25 % deoxycholic acid, 1 % NP-40, 10 mM EDTA) supplemented with protease and phosphatase inhibitor (PhosSTOP™ - Roche). Cells were lysed in ice with RIPA buffer for 20′ in orbital shaker at 250 rpm. Whole cell lysates were cleared 20′ at 12,000 x g. Protein concentration was quantified with Pierce™ BCA Protein Assay kit (Thermo Fischer Scientifics) and 20 ng of proteins were processed with 4X Loading Buffer and boiled 3′, then loaded in SDS-Page (NuPage 4–12 %) for protein separation. Proteins were blotted with Trans-BlotⓇ Turbo™ Transfer System on polyvinylidene difluoride (PVDF) membrane. Membrane was blocked with 5 % BSA in Tris Buffer Saline – 0,1 % Tween for 1 h at room temperature in orbital shaker at 50 rpm. Antibodies anti-p53 (#9282 CST Rabbit 1:1000) and anti-rabbit (#7074 CST HRP-linked 1:3000) were diluted in blocking solution.

### 2.6 HCoV-229E CPE assay

2*10^4^ BEAS-2B cells per well were seeded in transparent 96-well plate. After 24 h, cells were infected with HCoV-229E with a MOI of 0.06 in presence of compound or 0.1 % DMSO (untreated controls). Cells were incubated at 35 °C with 5 % CO_2_. After 72 h, 20 μl of 3-(4,5-dimethylthiazol-2-yl)-2,5-diphenyl-2H-tetrazolium bromide (MTT) (Sigma-Aldrich), dissolved in PBS at 7.5 mg/ml, were added to each well and incubated at 37 °C with 5 % CO_2_ for 1 h. Then the supernatant was removed, cells were lysed with 100 μl/well of 10 % 2-Propanol, 0.02 % Triton-X-100 (Sigma-Aldrich), 0.002 % HCl, and the absorbance was read at 570 nm with a plate reader Victor Nivo5 PerkinElmer.

### 2.7 HCoV-229E viral replication assay in MRC-5 cells

MRC-5 cells were seeded 1*10^5^ per well in 12-well plates and incubated overnight. 24 h later, cells were infected with a MOI of 0.2 and treated with indicated concentrations of compounds for 1 h at 35 °C with 5 % CO_2_ for 1 hour, then the inoculum was removed and substituted with compounds diluted in complete medium. 48 h post infection, RNA was extracted with TRIzol™ Reagent (Invitrogen), reverse transcribed and amplified using Luna universal one-step quantitative real-time PCR (RT-qPCR) kit (New England BioLabs), HCoV-229E Envelope protein mRNA expression levels (fw_primer: CGTCAGGGTAGAATACCTT; rv_primers: CCTGTGCCAAGATAAAA) were normalized to the level of GAPDH. Results are expressed as percentage of viral replication calculated with respect to the infected control. GC376 compound was used as positive control of viral inhibition (Hu et al., 2021). Compounds’ cytotoxicity was performed in parallel, 2*10^^4^ cells/well MRC-5 were seeded in 96 well plate, after 24 h cells were treated with decreasing concentrations of compounds. Cell viability was measured 48 h after treatment with MTT method as described above.

### 2.8 SARS-CoV-2 viral replication assay in BEAS-2B cells

BEAS-2B cells were seeded 3*10^5^ per well in 12-well plates and incubated overnight to reach 90 % confluency. 24 h later, cells were infected with a MOI of 0.2 and treated with indicated concentrations of compounds for 1 h at 37 °C with 5 % CO_2_ for 1 hour, then the inoculum was removed and substituted with compounds diluted in complete medium. 48 h post infection, RNA was extracted with TRIzol™ Reagent (Invitrogen), reverse transcribed and amplified using Luna universal one-step quantitative real-time PCR (RT-qPCR) kit (New England BioLabs), SARS-CoV-2 Spike protein mRNA expression levels (fw_primer: GTGTTTATTTTGCTTCCACT; rv_primer: GGCTGAGAGACATATTCAAAA) were normalized to the level of GAPDH. Results are expressed as percentage of viral replication calculated with respect to the infected control. GC376 compound was used as positive control of viral inhibition (Hu et al., 2021).

### 2.9 SARS-CoV-2 viral replication assay in Vero-E6 GFP

The SARS-CoV-2 viral replication assay in Vero-E6 GFP was performed as previously described (Corona et al., 2022). The inhibition of viral replication was calculated as percentage of virus-induced cytopathic effect on infected untreated controls. EC_50_ value was calculated with Prism 9. Version 9.1.2 via non-linear regression.

### 2.10 SARS-CoV-2 viral replication assay in Calu-3

The SARS-CoV-2 viral replication assay in Calu-3 cells was performed as previously described (Stefanelli et al., 2023). Compounds’ cytotoxicity was performed in parallel, 2*10^^4^ cells/well Calu-3 were seeded in 96 well plate, 24 h after cells were treated with decreasing concentrations of compounds. Cell viability was measured 48 h after treatment with MTT method as described above.

### 2.11 Immunofluorescence

BEAS2-B cells were seeded 5*10^4 cells per well in transparent 24 well plates. 24 h after seeding cells were treated with compound or 0.1 % DMSO (untreated controls) and infected with HCoV-229E with a MOI of 0.06 in presence of compound or 0.1 % DMSO (untreated controls) for 1 h at 35 °C, 5 % CO_2_. Then the inoculum was removed and replaced with compound or 0.1 % DMSO in complete medium. 6 h post infection, cells were fixed with 4 % PFA for 15′, washed three times with PBS, 7′ with glycine 100 mM, washed three times with PBS, permeabilized with 0,3 % Triton X-100 in PBS for 10′, blocked with 0,1 % Triton X-100, 5 % BSA in PBS for 60′, incubated 60′ with primary antibody Phospho-IRF3 (Ser396) (Invitrogen cat. 720012) diluted 1:2000 in blocking solution. Cells were washed three times with blocking solution and then incubated with secondary antibody Anti-Rabbit IgG - Atto 488 (Sigma-Aldrich cat. 18,772) diluted 1:500 for 60′ and washed three times with PBS. Post fixation was performed for 10′ with 4 % PFA, nuclei were stained with Hoechst 1μg/ml in PBS. Cells were washed three times with PBS and maintained in PBS for the image acquisition. Image acquisition was performed with the Cytation 5 Cell Imaging Multimode Reader (BioTek) and image analysis was performed with Gen5 Software for Imaging & Microscopy (BioTek).

### 2.12 Molecular modelling studies

*Ligand preparation.* Compounds global minimum conformation has been determined by molecular mechanics conformational analysis performed by Macromodel software version 9.2 (Mohamadi et al., 1990), considering Merck Molecular Force Fields (MMFFs) as force field and solvent effects by adopting the generalized Born/surface area (GB/SA) water implicit solvation model (Halgren, 1996; Kollman et al., 2000). The simulations were performed allowing 5000 steps Monte Carlo analysis with Polak–Ribier Conjugate Gradient (PRCG) method and a convergence criterion of 0.05 kcal/ (mol Å) was used. All the other parameters were left as default.

*Protein preparation.* The three-dimensional coordinates of the protein complexes were obtained from the Protein Data Bank (PDB) (Burley et al., 2019). Subsequently, the proteins were processed, and the hydrogen atoms were added, the multiple bonds and bond lengths were optimized using the algorithm implemented in Maestro's Protein Preparation Wizard using the default settings (Madhavi Sastry et al., 2013). The available 3D models were aligned, and the structure of the protein was analyzed in detail. In particular, the overlap of secondary structures and individual residues involved in the interaction with agonists.

*Docking experiments*. A Grid (30×30×30 Å) was centered on crystallized 6UKZ ligand. Several docking protocols were evaluated: Glide SP, Glide XP, Quantum Mechanics-Polarized Ligand (QMPL) SP and QMPL XP, Induced Fit docking (IF) SP, IF XP and seven ligands cocrystallized reported in the complexes with pdb code: 6UKU, 6UKW, 6UKZ, 6UKY, 6UL0, 6UKM, 7SII. The lower RMSD values were obtained considering Glide XP settings (Chung et al., 2009; Friesner et al., 2006; Halgren et al., 2004).

The new compound was then docked using the extra precision (XP) docking mode on the protein structure's generated grid and the Glide score was used to evaluate the final ligand-protein binding.

Complexes were analyzed with ligand interaction diagram in Maestro and Pymol (“The PyMOL Molecular Graphics System, Version 2.5.4 Schrödinger, LLC.,” 2024.).

*Druggable sites detection*. Sitemap was applied to the prepared protein to identify the druggable pockets. SiteScore, the relative scoring function was used to assess a site's propensity for ligand binding (Halgren, 2009a).

## 3 Results

### 3.1 Establishment of a reporter gene assay to select STING agonists

**Fig. 1:**
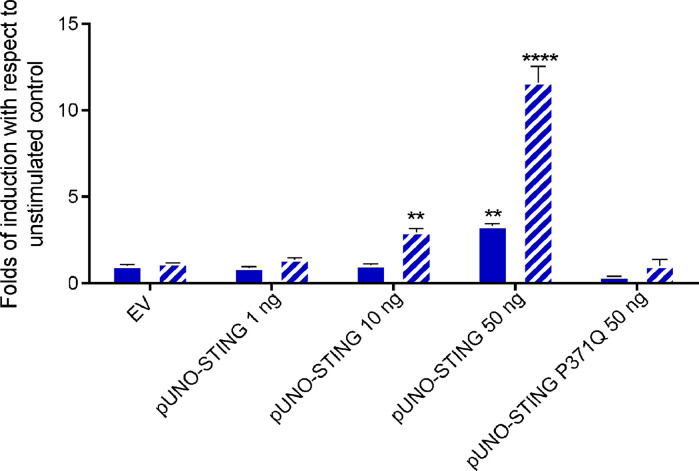
Establishment of pUNO-STING concentration for the IFN-I induction gene-reporter assay. HEK293T cells were transfected with pGL-IFN-β-luc (60 ng/well) and 1, 10, or 50 ng of pUNO-STING or 50 ng of Empty Vector (EV), or 50 ng of pUNO-STINGP371Q. 24 h after transfection, cells were stimulated with MSA (blue oblique stripes column) at 10 μM or equal volume of complete medium with DMSO (blue filled columns). 24 h after stimulation, cells were harvested, and luciferase activity was measured. Results are shown as pGL-IFN-β-luc folds of induction over not stimulated control in presence of EV. Values represent the mean ± SEM of three independent experiments based on triplicates. Asterisks indicate a significant difference obtained comparing EV-DMSO/pUNO-STING at different concentrations (two-way ANOVA test, n>=3) **p<0,01, **** p<0,001.

Given the STING involvement in DNA damage response, in most transformed cell lines there is an alteration of the cGAS-STING pathway. Hence, in the establishment of a reporter gene assay to test molecules potentially acting as STING agonists, it was considered more robust and controlled to use a cell line defective for STING, the Human Embryonic Kidney 293T (HEK293T) cell line, and transfecting it with a plasmid expressing exogenous STING, to then measure specifically the STING-dependent induction of the IFN-β gene (Miao et al., 2019; Suter et al., 2021; Thomsen et al., 2016).Therefore, HEK293T cells were transfected with a vector encoding wt STING and a reporter plasmid encoding the luciferase gene under the control of IFN-β promoter, as described in material and methods. The STING agonist MSA-2, was used as an induction control (Reus et al., 2020). Optimization of the assay led to identify the best background to MSA-2 induced signal ratio conditions (Fig. 1).

In addition, a mutated inactive form of STING was also used. In this mutant, STINGP371Q, the STING amino acid residue Pro371 is replaced with a Gln, which prevents STING from binding to TBK-1 and hence impedes the IFN-I induction. Indeed, MSA-2 was not able to induce the IFN-b promoter expression in the presence of the vector encoding STINGP371Q even at the highest plasmid tested concentration (Fig. 1).

### 3.2 STING dependent IFN-b promoter induction by BZF derivatives

**Fig. 2:**
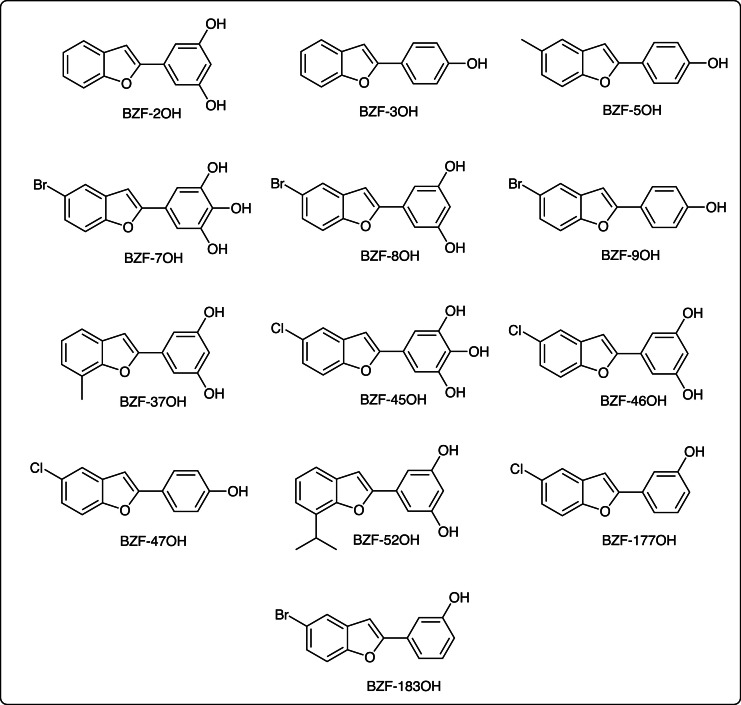
Chemical structure of the benzofurans derivatives.

**Fig. 3:**
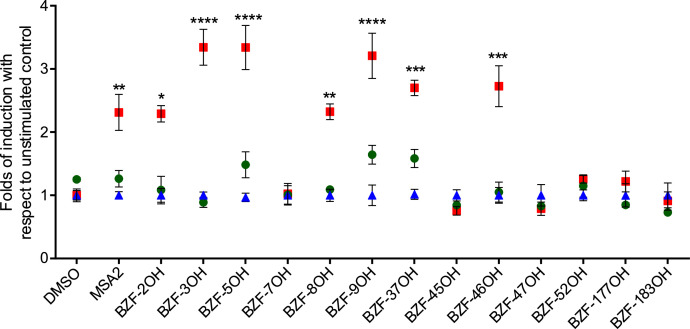
BZFs effect on STING-dependent IFN-β induction. HEK293T cells were transfected with pGL-IFN-β-luc and pUNO-STING (red square), or Empty Vector (blue triangle) or pUNO-STINGP371Q (green round) as described. 24 h after transfection, cells were treated with the indicated compounds at 10 μM concentration for 24 h and processed as described. Results are shown as pGL-IFN-β-luc folds of induction over not stimulated control in presence of EV. Values represent the mean ± SEM of three independent experiments based on triplicates. Asterisks indicate a significant difference with respect to the EV (two-way ANOVA test, n>=3) *p<0,05, **p<0,01, *** p<0,006, **** p<0,001.

Based on previous observations showing that benzothiophene (Pan et al., 2020) and benzimidazole derivatives (Zhu et al., 2021) are STING agonists, and the fact that the BZF scaffold is a bioisostere of both benzothiophene and benzimidazole substructures (Barillari and Brown, 2012; Brown, 2012), 13 BZF derivatives (Fig. 2) were selected to be evaluated in the above described assay to verify their ability to act as STING agonists. Results showed that, in the presence of wt STING, 7 out of 13 BZFs strongly induced IFN-β transcription (Fig. 3). In particular, compounds BZF-2OH, BZF-3OH, BZF-5OH, BZF-8OH, BZF-9OH, BZF-37OH and BZF-46OH significantly induced the IFN-I reporter gene expression (Fig. 3), while BZFs with three hydroxyl groups on the 2-phenyl ring (BZF-7OH and BZF-45OH) as well as those with only one hydroxyl in the meta position (BZF-177OH and BZF-183OH) were found to be inactive. In addition, compound BZF-52OH, which is substituted in position 7 by an isopropyl group, was inactive as compared to BZF-2OH, and compound BZF-47OH, which exhibits a chlorine atom in position 5, was also inactive as compared to compounds BZF-3OH, BZF-5OH and BZF-9OH. Overall, these results define structure-activity relationships for this chemical series.

To confirm that these BZFs induce the IFN-I expression STING-dependently, compounds were also tested in the presence of the inactive STINGP371Q. Results showed that the BZFs active on wt STING did not induce IFN-I expression in the presence of STINGP371Q, confirming their ability to act as STING agonists (Fig. 3).

### 3.3 BZFs do not induce DNA damage

**Fig. 4:**
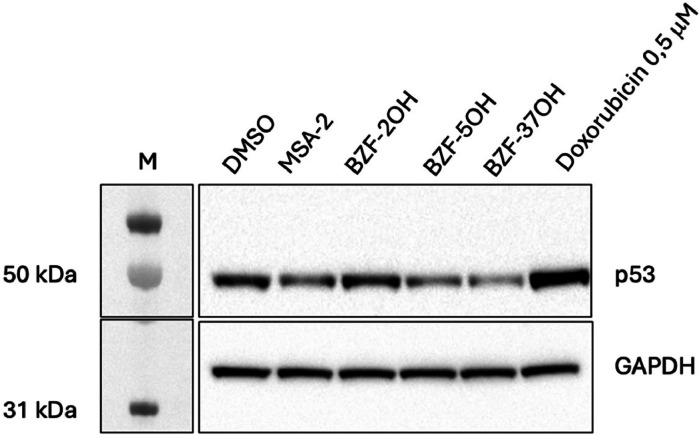
BZFs effect on p53 expression. HEK293T cells were treated with DMSO, MSA-2 and BZFs at 10 μM concentration or doxorubicin at 0.5 μM concentration for 24 h. Then cells were lysed and 20 ng of cell lysates was subjected to western blot. The experiment was repeated three times independently with similar results.

Given that the cGAS-STING pathway can be activated also by a cytosolic DNA release upon nuclear DNA damage, we wanted to exclude that BZF compounds could be genotoxic. Hence, the potential DNA damage induced by BZFs was assessed measuring the p53 levels in the presence of the compounds through western blot. The HEK293T cells were treated for 24 h with BZF-2OH, BZF-5OH and BZF-37OH, that were shown to induce the IFN-β reporter gene assay, using doxorubicin as genotoxyc positive control (Fig. 4) (Lin et al., 2018). Results showed that the p53 levels in the presence of the BZF compounds were comparable to the untreated control, excluding that BZFs could induce IFN-I expression through cytosolic DNA release.

### 3.4 Inhibition of HCoV-229E replication by BZF derivatives

To verify whether the BZFs induction of the IFN-I expression could lead to an antiviral effect, firstly we tested the BZF efficacy on the HCoV-229E replication in BEAS-2B cells, using compound GC376 as positive control (Seng et al., 2014). Results showed that among the seven compounds able to induce the IFN-β reporter gene expression, three were able to effectively inhibit HCoV-229E replication, namely BZF-2OH, BZF-5OH, BZF-37OH, with EC_50_ values in the μM range (Table 1). Differently, BZF-8OH and BZF-46OH were cytotoxic, while BZF-3OH and BZF-9OH were not able to inhibit viral replication even if they were not highly cytotoxic (Table 1). Of note, MSA-2 known as STING agonist was not able to inhibit viral replication and, indeed, at the best of our knowledge no report has been published showing an MSA-2 antiviral effect. To furher assess the compounds antiviral activity on HCoV-229E, the active BZFs were tested to evaluate their effect on the HCoV-229E replication in MRC-5 cells, confirming their antiviral activity in the same concentration range (Table 1).

**Table 1:** Effect of BZF derivatives on HCoV-229E replication.

Compound	HCoV-229E aEC_50_ (μM) in BEAS-2B (bSI)	BEAS-2B cCC_50_ (μM)	HCoV-229E dEC_50_ (μM) in MRC-5 (bSI)	MRC-5 eCC_50_ (μM)
**BZF-2OH**	14.7 ± 3.0 (4,8)	70.1 ± 7.0	9.6 ± 0.5 (>14.5)	> 100
**BZF-3OH**	> 30.1	> 30.1 ± 2.0	-	-
**BZF-5OH**	16.5 ± 1.4 (2.1)	36.5 ± 2.4	2.3 ± 1.9 (10.95)	25,2 ± 0,5
**BZF-7OH**	> 17.3	17.3 ± 4.4	-	-
**BZF-8OH**	> 8.7	8.7 ± 5.5	-	-
**BZF-9OH**	> 30.6	30.6 ± 10.2	-	-
**BZF-37OH**	17.5 ± 1.5 (4.6)	81.4 ± 4.2	9.0 ± 3.7 (3.5)	31.8 ± 1,8
**BZF-45OH**	> 8.2	8.2 ± 1.4	-	-
**BZF-46OH**	> 13.4	13.4 ± 0.7	-	-
**BZF-47OH**	> 18.9	18.9 ± 3	-	-
**BZF-52OH**	> 30	> 30 ± 3.0	-	-
**BZF-177OH**	> 25.2	25.2 ± 10.7	-	-
**BZF-183OH**	> 30	> 30 ± 2.5	-	-
**MSA-2**	> 30	> 30	-	-
**GC376**	0.12 ± 0.03 (>828)	>100	0.16 ± 0.07 (>625)	>100

a EC_50_, compounds’ concentration able to reduce by 50 % the HCoV-229E induced cytopathic effect in BEAS-2B cells, as compared to the untreated control.

b SI, selectivity index calculated as the ratio between CC_50_ and EC_50_ values.

c CC_50_, compounds’ concentration able to reduce by 50 % BEAS-2B cells viability.

d EC_50_, compounds’ concentration able to reduce by 50 % HCoV-229E viral RNA accumulation in MRC-5 cells, as compared to the untreated control.

e CC_50_, compounds’ concentration able to reduce by 50 % MRC-5 cells viability BEAS-2B values represent the mean ± SDs of three independent experiments based at least on 6 compounds concentrations in triplicate; MRC-5 values represent the mean ± SDs of two independent experiments in duplicate based on at least 4 concentrations in duplicate.

### 3.5 Inhibition of SARS-CoV-2 replication by BZF derivatives

To verify whether compounds BZF-2OH, BZF-5OH, BZF-37OH could also inhibit other HCoVs, we then tested their effect on SARS-CoV-2 replication. For better comparison of the results, we firstly wanted to assess the effects of the SARS-CoV-2 replication also in BEAS-2B cells. Given that it is known that SARS-CoV-2 replication is less efficient than HCoV-229E replication, we determined the replication efficiency observing a roughly 2-fold lower efficiency for SARS-CoV-2 replication with respect to HCoV-229E replication (data not shown). Considered that SARS-CoV-2 replication in BEAS-2B cells was sufficient for the evaluation of compounds effect, we tested them showing that BZF-2OH and the BZF-5OH were able to inhibit SARS-CoV-2 replication with EC_50_ values in the μM range, while compound BZF-37OH was unexpectedly inactive (Table 2). To furher assess the compounds antiviral activity on SARS-CoV-2 replication, BZFs effect was evaluated also using Calu-3 cells, in which SARS-CoV-2 has a higher replication efficiency with respect to BEAS-2B, showing an antiviral effect in the nM range for all three tested compounds (Table 2). Given that it has been reported that in Calu-3 cells infected by SARS-CoV-2 there is a strong cGAS/STING induction (up to 98 folds) as consequence to viral infection (Mösbauer et al., 2021; Zhou et al., 2021), the higher potency of SARS-CoV-2 inhibition observed in Calu-3 confirmed the mode of action of the compounds.

**Table 2:** Effect of BZF derivatives 2OH, 5OH and 37OH on SARS-CoV-2 replication.

Compound	SARS-CoV-2 BEAS-2B aEC_50_ (μM)	SARS-CoV-2 Calu-3 aEC_50_ (μM)	Calu-3 cCC_50_ (μM)	SARS-CoV-2 Vero E6 GFP bEC_50_ (μM)	Vero E6 GFP cCC_50_ (μM)
**BZF-2OH**	23.4 ± 1.1	0.23 ± 0.13	68.9 ± 2.9	> 9.5	9.5 ± 2.2
**BZF-5OH**	18.4 ± 1	0.24 ± 0.04	25.8 ± 7.7	> 76	76.5 ± 3.0
**BZF-37OH**	> 81.4	0.26 ± 0.05	>100	>100	>100
**GC376**	0.12 ± 0.003	0.0034 ± 0.0001	>100	0.63 ± 0.14	>100

a EC_50_, compounds concentration able to reduce by 50 % the SARS-CoV-2 viral RNA accumulation in BEAS-2B cells or Calu-3 cells supernatant, as compared to the untreated control.

b EC_50_, compounds concentration able to reduce by 50 % the SARS-CoV-2 CPE in Vero E6 GFP, as compared to the untreated control.

c CC_50_, compounds concentration able to reduce by 50 % Vero E6 GFP or Calu-3 cells viability. BEAS-2B and Vero E6 GFP values represent the mean ± SDs of three independent experiments based at least on 6 compounds concentrations in triplicate; Calu-3 values represent the mean ± SDs of two independent experiments in duplicate based at least on 6 compounds concentrations in duplicate.

To further confirm that compounds inhibition was indeed due to the IFN-I induction, we tested their inhibitory effect on SARS-CoV-2 replication in Vero E6 cells that are defective for IFN-I production. Importantly, as expected, BZFs did not inhibit SARS-CoV-2 in Vero E6 cells (Table 2), hence confirming that they act inducing IFN-I expression.

### 3.6 pIRF3 espression analysis

**Fig. 5:**
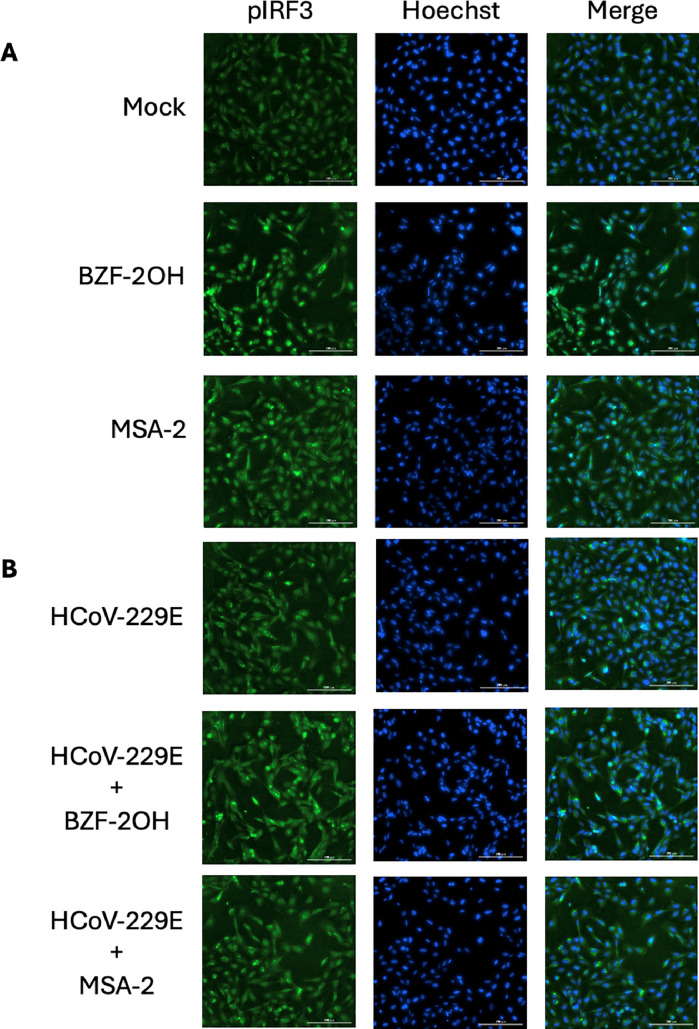
Effect of BZF-2OH and MSA-2 on IRF3 phosphorylation in mock and infected cells. Immunofluorescence of BEAS-2B cells uninfected (A) and infected (B) with HCoV-229E MOI 0.06 in presence and absence of 10 μM BZF-2OH or MSA-2. 6 h post infection and treatment cells were fixed and stained as described. The images are representative of two independent experiments. Nuclei are stained in blue and S396 pIRF3 is stained in green. Images were acquired at 20× magnification. Scale bar = 200 μm. (For interpretation of the references to color in this figure legend, the reader is referred to the Web version of this article.).

Phospho-IRF3 is a main interactor of the cGAS-STING pathway, hence, to further verify that BZFs act as STING agonists, we wanted to evaluate whether they trigger the IRF3 phosphorylation. To this aim, IRF3 phosphorylation was evaluated in BEAS-2B uninfected cells (Fig. 5A) as well as in BEAS-2B infected by HCoV-229E (Fig. 5B), in absence and presence of BZF-2OH or MSA-2. MSA-2 was used as control of STING mediated IRF3 phosphorylation (Pan et al., 2020). Images were taken 6 h post infection and subpopulation analysis was performed with the Gen5 software (BioTek).

The subpopulation analysis showed that pIRF3 nuclear and cytoplasmic levels in BEAS-2B infected cells increased by 2.3- and 13-fold, respectively with respect to the uninfected control cells. In presence of BZF-2OH pIRF3 nuclear and cytoplasmic levels increased by 9.4- and 32.2-fold, respectively, as compared to the uninfected control cells. Similarly, in presence of MSA-2 pIRF3 nuclear and cytoplasmic levels increased by 3.6- and 21-fold, respectively, as compared to the uninfected control cells. Interestingly, the comparison of the effects of BZF-2OH in infected BEAS-2B, showed that the pIRF3 levels both nuclear and cytoplasmic are reduced with respect to uninfected cells, since in infected cells BZF-2OH induces a 2.2- and 19.1- fold increase of nuclear and cytoplasmic pIRF3 induction, respectively. Of note, the comparison of the effects of MSA-2 in infected BEAS-2B, showed that the pIRF3 levels both nuclear and cytoplasmic are even more reduced with respect to uninfected cells, since in infected cells MSA-2 led to a 1.1- and 13- fold increase of nuclear and cytoplasmic pIRF3 induction, respectively. Overall, these results demonstrate that BZF-2OH acts as STING agonist and show that viral infection (probably due to innate immunity evasion mechanisms) reduces the effect of STING induction by both BZFs and MSA-2 in different degrees. The fact that MSA-2 does not increase IRF3 phosphorylation in BEAS-2B infected cells may explain its lack of antiviral effect.

### 3.7 Docking studies

**Fig. 6:**
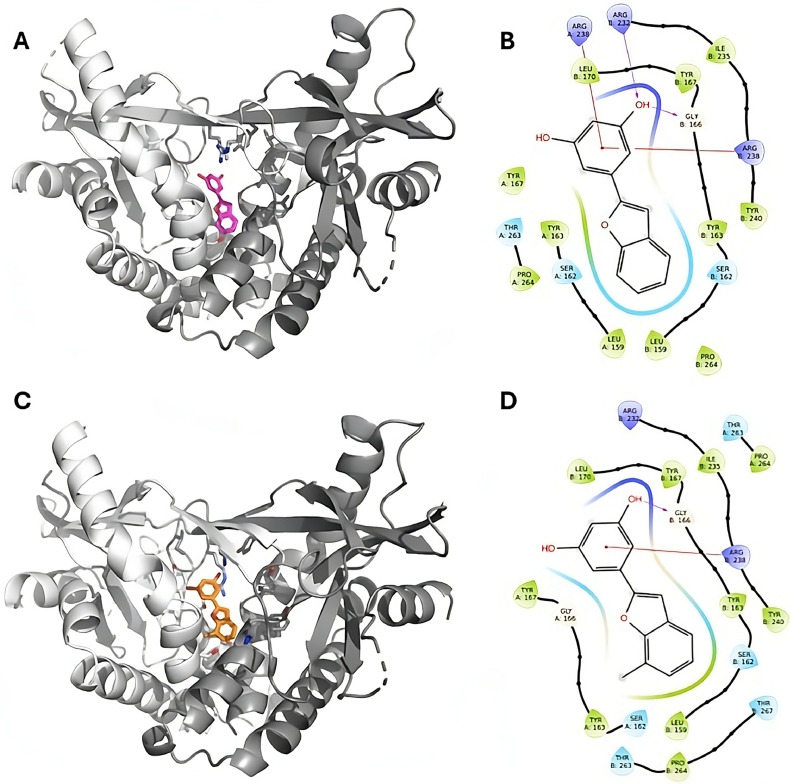
Putative binding mode of BZF-2OH and BZF-37OH. Panels A, C: putative 3D representation of ligands binding mode to STING: A-chain, in light grey, and B, in dark grey; panels B, D: corresponding 2D representation of the interactions.

**Fig. 7:**
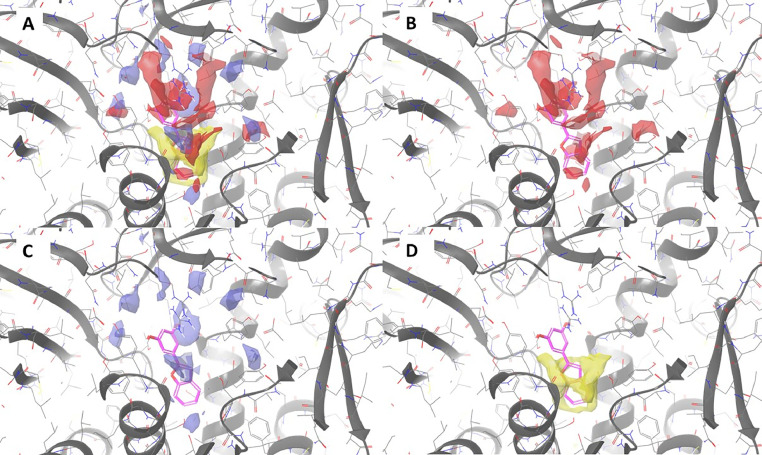
SiteMap Analysis of STING binding pocket. Panel A: druggable site identified by Sitemap and relative maps; panel B: hydrogen-bond acceptor map in red. Panel C: hydrogen-bond donor map in violet. Panel D: hydrophobic map in yellow. The best docked compound, BZF-2OH, it is shown in magenta sticks.

To gain further insights on BZF interaction with STING, the most promising and selective compounds, BZF-2OH and BZF-37OH, were then considered for molecular docking studies to predict their putative binding mode considering the STING crystal structure with pdb code 6UKZ (Pan et al., 2020). The docking protocol was validated through re- and cross-docking, while taking into account the crystallographic data of seven ligands. The docking predicted binding mode of ligands to STING extracellular cavity is shown in Fig. 6. A further analysis was performed applying Sitemap to understand how the BZF derivatives could be optimized. The analysis highlights areas within the BZFs binding pocket which are suitable for occupancy by ligands with hydrogen bond acceptors (red maps), donors (violet) or hydrophobic groups (yellow maps) (Fig. 7) (Halgren, 2009b). The differentiation of the various binding site sub-regions allows a quick assessment of a ligand's complementarity. We observed that both donor (violet) and acceptor maps (in red) are well-represented (Fig. 7B).

## 4 Discussion

Ongoing viral evolution, climate change and spillover events represent a major health issue worldwide. Hence, innovative therapeutic approaches are required to effectively counteract and control viral spread, also considering novel potential epidemics. On the path to the discovery and development of broad-spectrum antiviral agents, one possibility is to target cellular proteins to trigger a strong innate immune response capable of blocking viral replication. STING has been identified as a potential target for this strategy due to its central role in the innate immune response (Deng et al., 2014b; Maringer and Fernandez-Sesma, 2014; Unterholzner and Dunphy, 2019b; Woo et al., 2014b).

BZFs have been shown to posses several biologically interesting activities, including anticancer and antiviral activities (Delogu et al., 2022; Duncan et al., 2021; Era et al., 2020; Fais et al., 2019; Ferino et al., 2013; Galal et al., 2009; Geldenhuys et al., 2012; Jiang et al., 2008; Kumar et al., 2018; Pisani et al., 2013; Van Dyk et al., 2015; Xu et al., 2019). Firstly, BZFs can be isolated from natural products such as: *Machilus glaucescens, Ophryosporus charua, Ophryosporus lorentzii, Krameria ramosissima, Ammi majus L.,* and *Zanthoxylum ailanthoidoland* presenting antihyperglycemic, analgesic, antiparasitic, antimicrobial, antitumor and kinase inhibitory properties (Khanam and Shamsuzzaman, 2015); secondly, BZFs are included in commonly used medicines all of them showing a good bioavailability among the species tested: amiodarone, ailanthoidol, bufurarol β-adrenoceptor antagonist, opioids (i.e.: codeine, oxymorphone, morphine) (Nevagi et al., 2015; Xu et al., 2019).

Known STING agonists often show a moiety that mimic the purine bases of the substrate: e.g. benzothiophene derivatives (MSA-2) and benzoimidazole derivatives (di-ABZI). Hence, BZF derivatives were tested as promising scaffold for the design of novel STING agonists. In fact, typical isosteric substitutions are -S- with -O- and – N=. with-CH= (Barillari and Brown, 2012; Brown, 2012).

To study potential STING agonists, we firstly established a novel luciferase gene reported cell-based assay that was then used to test thirteen BZFs and then identified seven BZFs that are able to significantly induce IFN-β driven luciferase expression in presence of wt STING. The lack of BZF induction of IFN-b expression in the presence of mutant and inactive STINGP371Q confirms the STING engagement in their mode of action.

Antiviral assay showed that BZF derivatives are able to inhibit HCoVs replication, namely HCoV-229E and SARS-CoV-2, in different cell lines. The different potency of inhibition of viral replication observed in the different cell lines are probably to be linked to the different levels of STING expression, activation upon viral infection and inhibition by viral infection. In fact, the difference in antiviral efficacy among some BZF derivatives as well as the lack of antiviral effects of the known STING agonist MSA-2 point to the need of further investigation of their interplay with viral proteins that may reduce their ability to act as STING agonists.

The hypothesis that BZF derivatives inhibit viral replication by active as STING agonist is clearly demonstrated by the lack of induction of luciferase production using mutant STINGP371Q, the lack of SARS-CoV-2 inhibition in Vero E6 cells and the induction of IRF3 phosphorylation and nuclear translocation in cells treated with BZF-2OH in both infected and uninfected cells. Again, the lack of increase of the IRF3 phosphorylation and nuclear translocation observed in the presence of MSA-2 in 229E infected cells seems to suggest that viral infection can alter MSA-2 efficacy and requires further investigation to be better understood.

Docking experiments allowed to predict the binding mode of best compounds BZF-2OH and BZF-37OH. The complexes are stabilized by hydrogen bond interactions (with Arg312 and Gly166, for BZF-2OH and Gly 166, for BFZ-37OH) and strong cation-π interactions between the ligands and the Arg238 residues of the STING dimer. Furthermore, several van del Walls interactions, including Leu159, Tyr163, Tyr167, Leu 170, Ile235, Tyr240, and Pro264, from both dimer chains, also contributed. The binding mode analysis helps to understand the SAR for this chemical series. Although the mono substitution of the benzofuran ring is relatively well tolerated the steric hindrance of a larger substituent is associated with a loss of activity, as in BZF-52OH in position 7. SAR also suggests that the presence of three OH groups on the 2-phenyl ring as in BZF-7OH and BZF-45OH is detrimental to the compounds activity. However, given the overall druggable site, results suggest that it is possible to increase the compound size, and this could lead to increase selectivity. Indeed, cGAMP and some known agonists such as di-ABZI (Ramanjulu et al., 2018) and the MSA-2 dimers (Pan et al., 2020) are reported to occupy this large area. Altogether, this might help to increase the activity of studied molecules possibly reducing their toxicity.

## 5 Conclusions

Overall, the cellular testing combined with in silico studies demonstrated that some BZF derivatives are selective STING agonist, able to induce the innate immune response thus inhibiting HCoVs replication in different cell lines. The presented data indicate that BZF derivatives can be used as chemical scaffold to target STING and develop broad-spectrum antivirals.

## CRediT authorship contribution statement

**A. Paulis:** Writing – review & editing, Writing – original draft, Investigation, Conceptualization. **A. Onali:** Writing – original draft, Investigation. **P.O. Vidalain:** Writing – review & editing, Conceptualization. **V. Lotteau:** Writing – review & editing, Supervision. **C. Jaquemin:** Writing – review & editing. **A. Corona:** Writing – review & editing, Supervision. **S. Distinto:** Writing – original draft, Investigation, Conceptualization. **G.L. Delogu:** Writing – original draft, Investigation, Conceptualization. **E. Tramontano:** Writing – review & editing, Writing – original draft, Supervision, Funding acquisition, Conceptualization.

## Declaration of competing interest

The authors declare that they have no known competing financial interests or personal relationships that could have appeared to influence the work reported in this paper.

## Data Availability

Data will be made available on request.
